# New Limits for the Period of Lactation

**Published:** 1843-10

**Authors:** 


*New Limits for the period of Lactation.*—Dr. Loudon, in a recently published work on population, has named the period of three years as that during which the infant should receive its nourishment by lactation. His argument is the following: "Of all mammiferæ, none is so long as man in being able to sustain itself upon its legs, in reaching the age of puberty and adult age, and, taking his size into consideration, none is endowed with a longevity comparable with his. Man is completely developed at 21; few men have lived till 150. Now, if we divide the first number by 7, and the second by 49, we shall find that the period of lactation for the human race ought to be three years."—*Med. Examiner*, from *Provin. Med. Journ.*, June 34, 1843.

